# Development of an Electronic Medical Record–Based Score for Heart Failure Prediction in Cancer Survivors

**DOI:** 10.1016/j.jacadv.2025.102129

**Published:** 2025-09-11

**Authors:** Cheng Hwee Soh, Lena Nguyen, Anna Chu, Agus Salim, Husam Abdel-Qadir, Thomas H. Marwick

**Affiliations:** a Imaging Research Laboratory, Baker Heart and Diabetes Institute, Melbourne, Australia; b Baker Department of Cardiometabolic Health, University of Melbourne, Melbourne, Australia; c Baker Department of Cardiovascular Research, Translation and Implementation, La Trobe University, Melbourne, Australia; d Institute for Clinical Evaluative Sciences (ICES), Toronto, Ontario, Canada; e Menzies Institute for Medical Research, Hobart, Australia

**Keywords:** cardio-oncology, cardiovascular prevention, cardiovascular toxicity

## Abstract

**Background:**

Awareness of heart failure (HF) as a long-term complication of cancer has led to an interest in HF surveillance among survivors. However, existing HF risk scores are not tailored for survivors and not designed for the use in electronic medical records (EMRs) or administrative data sets where clinical data such as blood pressure and pathology results are often unavailable.

**Objectives:**

The objective of the study is to develop a cancer-specific incident HF risk score suitable for screening in EMR or administrative data sets.

**Methods:**

The cancer-specific HF prediction from EMRs in survivor health care (CHERISH) risk score was developed from risk variables identified in 16,191 cancer survivors (mean 61 years; 59.5% female) derived from the UK Biobank. External validation was conducted in a population-based Ontario cohort (n = 446,096; mean 67 years; 53.9% female). HF risk classification with CHERISH was compared against the ARIC (Atherosclerotic Risk In Community)-HF score using area under the curve (AUC).

**Results:**

The CHERISH score incorporates 11 clinical variables—age, years since cancer diagnosis, coronary heart disease, arrhythmia, myocardial infarct, diabetes, hypertension, leukemia, non-Hodgkin lymphoma, lung cancer, and breast cancer. CHERISH demonstrated strong prediction of 10-year HF incidence during internal validation (AUC: 0.829), exceeding ARIC-HF (AUC: 0.697; *P* < 0.001). In external validation, CHERISH showed an AUC of 0.721 in predicting 10-year HF incidence, compared to an AUC of 0.751 (*P* < 0.001) with ARIC-HF.

**Conclusions:**

The integration of the CHERISH score into EMR systems may provide large-scale, automated HF risk assessment in cancer survivors, using routinely collected clinical data.

Advances in cancer treatments over recent decades have increased the numbers of survivors and their lifespan.^1^ However, these benefits have been at the cost of increased rates of morbidity,^2^ with a growing epidemic of cardiovascular diseases, especially heart failure (HF) among survivors.^3^ Current European Society of Cardiology guidelines do not provide a standardized approach in managing the long-term HF risk among cancer survivors.^4^ However, a means of clinically assessing the risk of future HF incidence in large groups of survivors at risk of cancer therapy–related cardiac dysfunction could allow those most at risk to be investigated further or even start cardioprotective therapy.^5^

Although there are numerous tools for assessing the risk of incident HF, none of them specifically apply to cancer survivors, who have both incident HF risk and high levels of competing risks. A cancer-specific risk assessment tool has been developed by the Cardio-Oncology Study Group from the Heart Failure Association of the European Society of Cardiology and the International Cardio-Oncology Society (HFA-ICOS) to classify low, medium, high, and very high risk groups at the time of chemotherapy.^6^ However, there are 2 problems with applying the HFA-ICOS score during survivorship. First, it requires details of cancer therapy (which are often inaccessible once the patient has been discharged to community care), and second, the value of this test for predicting incident HF in survivorship is unproven. Therefore, we sought to develop and validate a simple clinical risk tool that is easily applicable: 1) at the first point-of-contact in a primary care setting; 2) in the electronic medical record (EMR); and 3) health authorities in administrative data sets to predict the future HF incidence among cancer survivors.

## Methods

### Study design and setting

The UK Biobank is a large (n = 487,177), population-based, prospective cohort study of adult participants recruited from the United Kingdom from 2006 to 2010.^7^ Participants underwent a series of baseline assessment visits, including questionnaires and brief verbal interview, physical measurement, fitness test, and electrocardiogram. Blood, urine, and saliva samples were also collected.

The external validation phase of this study used linked administrative data sets in the Canadian province of Ontario. Using the Ontario Cancer Registry (OCR),^8^ all cancer survivors with a cancer diagnosis before January 1, 2014, were identified. The OCR is a provincial database which records data on individuals diagnosed with almost all malignancies (except for nonmelanoma skin cancers) and is estimated to capture more than 95% of all diagnoses in the province, The OCR was then linked to the Canadian Institute of Health Information Discharge Abstract Database, National Ambulatory Care Recording System, and the Ontario Health Insurance Plan Physician Billing Claims database using unique encoded identifiers and analyzed at the Institute for Clinical Evaluative Sciences. Exclusion criteria included: 1) death date on or before index date (January 1, 2014); 2) ineligible for the Ontario Health Insurance Plan at any point in the 5-year period before index date; 3) resided in a long-term care home in the 5-year period before index date; 4) age <40 or >105 years on index date; 5) diagnosis of HF before/on index date; and 6) not residing in Ontario, Canada, at index date.

### Ethical approval

This study was conducted under the application ID of 55469 for the UK Biobank database (version 3.4). The UK Biobank was approved by the North West–Haydock Research Ethics Committee (16/NW/0274) and followed the guidelines outlined in the Declaration of Helsinki.

For the external validation, the use of data in this project was authorized under section 45 of Ontario's Personal Health Information Protection Act, and does not require review by a Research Ethics Board.

### Cancer diagnoses

Cancer diagnoses were determined based on documentation in the 10th version of the International Classification of Diseases (ICD-10) code (Field ID 40006).^9^ Cancer types were determined from C00 to C97 and D00 to D48, with exclusion of some cancer types (melanoma and other malignant neoplasms of skin, malignant neoplasms of mesothelial and soft tissue, in situ neoplasms, benign neoplasms, and neoplasms of uncertain or unknown behavior).

### Clinical characteristics

In the UK Biobank study, participant characteristics, including but not limited to age (Field ID 33), sex (Field ID 31), heart rate (Field ID 102), systolic (Field ID 4080) and diastolic (Field ID 4079) blood pressure, smoking status (Field ID 20116), and cholesterol level (Field ID 30690, 30460 and 30790), were collected during baseline assessment. Diagnoses of comorbidities, such as diabetes, hypercholesterolemia, coronary heart disease, history of acute myocardial infarction, and chronic kidney disease, were determined as per documentation in ICD-10 code. In addition, documentation of the medications for hypertension, cholesterol, and diabetes were available (Field ID 6177 and 6153). UK Biobank participants with any missing data (mainly heart rate, blood pressure, and cholesterol levels) were excluded from the analyses.

Most of these characteristics were also available in the external validation database. Specifically, data on participants' age at index date (January 1, 2014), sex, years since cancer diagnosis, cancer types, hypertension, diabetes, history of acute myocardial infarct, coronary heart disease, and arrhythmia were extracted. Participants with any missing data (primarily cancer types and years since cancer diagnosis) were also excluded from the external validation.

### HF diagnoses

HF incidence was determined based on the documentation of diagnosis with the ICD-10 code—I50. Date of HF diagnosis was also extracted, and the duration from date of consent to HF diagnosis was calculated. Participants who had a history of HF before consent were excluded from this study. In the external validation data set, HF was determined using a validated algorithm based on hospital, emergency department, and physician billing claim records.^10^

### Comparator HF prediction models

The ARIC (Atherosclerosis Risk In Community Studies)-HF risk score was used as a comparator for HF prediction in internal and external validation. It was computed among a subpopulation of survivors with complete primary care data, which was required to provide data on smoking status, blood pressure, heart rate, and body mass index (BMI) (Supplemental Figure 1).^11^

### Statistical analyses

Baseline characteristics are reported using descriptive statistics. All continuous variables are reported as mean ± SD with categorical variables as count (percentage). The start date for each participant was determined by the date of consent to the UK Biobank study, an average of 7.4 years from cancer diagnosis. The overall HF incidence, per 1,000 people-year, was reported among UK Biobank survivors from each cancer type. In addition, after adjusting for age and sex, the association between each cancer diagnosis and HF incidence was evaluated via the Cox regression analysis, reported in HR and 95% CI. Noncancer survivors were selected as the reference group. To develop and validate a new incident HF risk score, specifically for cancer survivors, the UK Biobank database of all survivors was split in a 7:3 ratio for training and testing. The statistical differences in clinical characteristics between training and testing data set were analyzed using *t*-tests (for continuous variables) and chi-square tests (for categorical variables). Internal model performance was evaluated in the independent testing set to assess discrimination and calibration. As the primary aim was to develop a pragmatic and scalable clinical risk score, optimism correction of beta coefficients via bootstrapping was not performed.

Due to the high dimensional nature of the data set and extensive list of diseases from the ICD-10 code documentation, we determined that a random forest model was most suitable for the variable selection of the new risk score. The model was applied in the training data set^12^ to identify variables associated with HF incidence among cancer survivors. These clinical characteristics and cancer types were ranked from highest to lowest in their mean decrease in Gini coefficients to identify the key variables for HF prediction. Based on this process, characteristics with high importance in HF prediction (mean decrease in Gini coefficients >10) were selected in the multivariable Fine and Gray competing risk regression model with HF incidence as the primary outcome of interest and death as the competing risk. The rationale for combining these 2 methods was to leverage the RF model's robust capability to handle complex interactions and nonlinear relationships among high-dimensional variables, while ultimately providing interpretable hazard estimates through the Fine and Gray model, which appropriately accounts for competing risks. Subsequently, a simplified version of the regression model was constructed after collinearity checking (variance inflation factors >10) and replacing certain variables using clinicians' discretion based on background knowledge (variables added were diabetes, hypertension, non-Hodgkin's lymphoma, leukemia, lung cancer, breast cancer, and years since cancer diagnosis).^13^ Pairwise interaction terms among the top 15 predictors were also evaluated in the regression model, of which no interaction achieved *P* < 0.01 (0.01 was selected due to reduced power and multiple comparisons), indicating negligible interaction effects. The regression coefficients for each variable were used to develop the cancer-specific HF prediction from EMRs in survivor health care (CHERISH) risk score for at 3-, 5-, and 10-years of survivorship. The functional form of continuous predictors (BMI, blood pressure, and cholesterol) was assessed by comparing models with linear terms vs restricted cubic splines using the Akaike Information Criterion. Variables were modeled linearly when spline terms did not substantially improve model fit (Supplemental Table 1). Age and years since cancer diagnosis demonstrated improved model fit when modeled using restricted cubic splines compared to linear terms. These variables were subsequently categorized based on clinical judgment to reflect meaningful survivorship phases and the associated incident HF risk while retaining model simplicity for routine application. The calibration slope of the final model in the testing data set was reported.

The newly developed CHERISH risk score (https://baker-biostats.shinyapps.io/CHERISH/) was then used to estimate the risk of HF in every participants in the validation databases.^14^ The performance of the CHERISH risk score in predicting HF incidence at 3-, 5-, and 10-years were evaluated using the area under the curve (AUC) and the Brier score. Youden index was applied in the training data set to determine the ideal cutoff value for each tool by maximizing the difference between sensitivity and specificity in the receiver operating characteristics (ROC) curve.^15^ The cutoff value is the model-predicted probability threshold above which an individual is classified as “high risk” for HF; any individual whose predicted 3-, 5-, and 10-year HF probability exceeds this threshold is deemed to have a positive prediction. The predictive performance of ARIC-HF was also reported in the UK Biobank testing data set. Decision curve analysis was performed to evaluate the clinical utility of the CHERISH risk score by comparing its net benefit across a range of threshold probabilities against the default strategies of treating all patients or treating none.

Within the external validation database, the CHERISH risk score was calculated for all included survivors with primary care data. The cohort was then divided into deciles of the 10-year CHERISH risk score and a calibration plot was generated by comparing the mean model-predicted incident HF risk at 3-, 5-, and 10-year to the observed cumulative incidence for each risk score decile.

The 2015 Transparent reporting of a multivariable prediction model for individual prognosis or diagnosis checklist for prediction model development was used to ensure reporting quality (Supplemental Material). A *P* value of <0.05 was considered statistically significant. Analyses were conducted in R (R Foundation for Statistical Computing) or SAS (version 9.4; SAS Institute).

## Results

Cancer diagnoses were documented among 43,720 of the 484,533 participants consented to the UK Biobank (Supplemental Figure 1). After excluding participants with skin cancer and other cancer types (n = 18,686), as well as those with missing data (n = 8,843), 16,191 survivors were included in the analyses. Table 1 shows the HF incidence and adjusted HR for each cancer type. Overall, HF (total incidence n = 1,592, 9.8%) was most prevalent among survivors of malignant neoplasms of lymphoid, hematopoietic and related tissue (7.42 per 1,000 person-years), urinary tract (5.70 per 1,000 person-years), and respiratory and intrathoracic organs (5.04 per 1,000 person-years). The corresponding HRs, adjusted for age and sex, for the 3 cancer types were 3.16 (95% CI: 2.93-3.42), 1.64 (95% CI: 1.49-1.80), and 2.73 (95% CI: 2.48-3.02).

**Table 1:** Cancer Diagnoses of the UK Biobank Participants and Its Association With HF Incidence Compared to Noncancer Participants

	Total (N = 25,034)	Age (y)	HF Incidence, Per 1,000 People-Year	HR (95% CI)
Malignant neoplasms of lip, oral cavity, and pharynx	562 (2.24)	59.2 ± 7.0	3.41	**1.48 (1.18-1.85)**
Malignant neoplasms of digestive organs	3,541 (14.14)	62.2 ± 6.1	3.84	**1.57 (1.46-1.69)**
Malignant neoplasms of respiratory and intrathoracic organs	1,157 (4.62)	62.3 ± 6.0	5.04	**2.73 (2.48-3.02)**
Malignant neoplasms of bone and articular cartilage	82 (0.33)	59.7 ± 7.4	3.35	1.10 (0.53-2.31)
Malignant neoplasms of breast	7,377 (29.47)	59.8 ± 6.8	2.37	**1.39 (1.27-1.53)**
Malignant neoplasms of female genital organs	1,636 (6.54)	60.2 ± 7.2	2.43	**1.66 (1.42-1.95)**
Malignant neoplasms of male genital organs	3,203 (12.79)	63.3 ± 5.7	4.05	**0.84 (0.77-0.91)**
Malignant neoplasms of urinary tract	1,853 (7.40)	62.9 ± 5.8	5.70	**1.64 (1.49-1.80)**
Malignant neoplasms of eye, brain, and other parts of central nervous system	299 (1.19)	58.5 ± 7.9	2.51	**1.95 (1.48-2.57)**
Malignant neoplasms of thyroid and other endocrine glands	298 (1.19)	57.8 ± 7.8	1.51	**1.95 (1.48-2.57)**
Malignant neoplasms of ill-defined, secondary, and unspecified sites	2,848 (11.38)	60.8 ± 6.9	3.47	**2.33 (2.12-2.55)**
Malignant neoplasms, stated or presumed to be primary, of lymphoid, hematopoietic, and related tissue	1,953 (7.80)	60.2 ± 7.5	7.42	**3.16 (2.93-3.42)**
Malignant neoplasms of independent (primary) multiple sites	379 (1.51)	63.1 ± 5.7	3.72	**1.32 (1.06-1.64)**

Cancer diagnoses were classified according to the tenth version of the International Classification of Diseases code. The start date for each participant was determined by the date of consent and participants were determined as survivors or noncancer controls based on cancer diagnosis before consent. Time to HF incidence was defined from consent date to HF occurrence. Participants' ages are reported in mean ± SD. The associations between cancer diagnoses and HF incidence were evaluated via multivariable Cox regression analyses, adjusted for age and sex. Noncancer participants were set as reference groups for the Cox regression. **Bold** values indicate statistical significance (*P* < 0.05).

HF = heart failure.

### Development of the CHERISH risk score

A 7:3 split of the database resulted in a training data set of 11,384 and a testing set of 4,807 cancer survivors. Most of the characteristics were not significantly different between the 2 data sets (Table 2). The overall HF incidence was 3.55 and 3.49 per 1,000 person-years in the UK Biobank training and testing data sets, respectively.

**Table 2:** Baseline Clinical Characteristics of the UK Biobank Participants and Ontario Residents With Primary Care Data

	UK Biobank			Ontario Residents (n = 7,921)
	Training Data Set (n = 11,384)	Testing Data Set (n = 4,807)	*P* Value	
Age (y)	60.99 ± 6.81	61.00 ± 6.75	0.92	67.24 ± 11.67
Female, n (%)	6,751 (59.30)	2,881 (59.93)	0.47	4,516 (57.0%)
Years since cancer diagnosis (y)	7.43 (6.69)	7.44 (6.79)	0.91	9.07 (8.13)
Ethnicity, n (%)			0.84	
White	11,017 (96.78)	4,642 (96.57)		-
Mixed	44 (0.39)	24 (0.50)		-
Asian	96 (0.84)	42 (0.87)		-
Black	115 (1.01)	57 (1.19)		-
Chinese	20 (0.18)	6 (0.12)		-
Other	57 (0.50)	23 (0.48)		-
Body mass index (kg/m^2^)	27.54 ± 4.81	27.42 ± 4.77	0.13	28.05 ± 5.54
Systolic blood pressure (mm Hg)	140.10 ± 18.95	140.34 ± 19.33	0.45	129.59 ± 17.65
Diastolic blood pressure (mm Hg)	82.12 ± 10.02	82.35 ± 10.17	0.19	75.18 ± 12.24
Heart rate (bpm)	70.73 ± 11.54	71.25 ± 11.96	**0.011**	75.55 ± 14.56
Smoking status, n (%)			0.93	
Never smoke	5,565 (48.88)	2,344 (48.76)		3,997 (50.5)
Previous smoker	4,722 (41.48)	2,007 (41.75)		3,097 (39.1)
Current smoker	1,097 (9.64)	456 (9.49)		827 (10.4)
Total cholesterol (mmol/L)	5.71 ± 1.19	5.74 ± 1.19	0.29	-
High-density lipoprotein cholesterol (mmol/L)	1.46 ± 0.39	1.46 ± 0.40	0.73	-
Low-density lipoprotein cholesterol (mmol/L)	3.56 ± 0.90	3.57 ± 0.90	0.35	-
Comorbidities				
History of acute myocardial infarct, n (%)	348 (3.06)	159 (3.31)	0.43	573 (7.2%)
Arrhythmia, n (%)	249 (2.19)	124 (2.58)	0.14	1,889 (23.8%)
Coronary heart disease, n (%)	592 (5.20)	275 (5.72)	0.19	2,594 (32.7%)
Valvular heart disease, n (%)	134 (1.18)	51 (1.06)	0.58	-
Cardiomyopathy, n (%)	34 (0.30)	17 (0.35)	0.68	-
Angina, n (%)	344 (3.02)	133 (2.77)	0.41	-
Diabetes, n (%)	718 (6.31)	305 (6.34)	0.96	1,669 (21.1%)
Chronic kidney disease, n (%)	83 (0.73)	30 (0.62)	0.53	-
Medications				
Blood pressure medication, n (%)	3,051 (26.80)	1,260 (26.21)	0.45	-
Cholesterol medication, n (%)	2,406 (21.13)	1,019 (21.20)	0.95	-
Insulin injection, n (%)	139 (1.22)	59 (1.23)	0.99	-
Quality of life, n (%)			**0.002**	
Poor	951 (8.35)	440 (9.15)		-
Fair	3,237 (28.43)	1,478 (30.75)		-
Good	6,156 (54.08)	2,453 (51.03)		-
Excellent	1,040 (9.14)	436 (9.07)		-
Heart failure incidence, per 1,000 people-year	3.55	3.49	0.85	15.1

Values are mean ± SD unless otherwise indicated. **Bold** values indicate statistical significance (*P* < 0.05).

The random forest analysis was used to identify relevant variables from the training data set. These characteristics included BMI (mean decrease in Gini 63.57; HR: 1.08; 95% CI: 1.07-1.09), age (50.98; HR: 1.10; 95% CI: 1.09-1.11), low-density lipoprotein cholesterol (47.84; HR: 0.68; 95% CI: 0.64-0.72), heart rate (47.55; HR: 1.01; 95% CI: 1.01-1.02), and total cholesterol (46.86; HR: 0.72; 95% CI: 0.69-0.75) as the 5 of the most important variables in HF prediction based on the random forest model. Supplemental Table 2 also demonstrates the univariable Fine and Gray competing risk regression analyses for each of the characteristics. Some of the key comorbidities were shown to be significantly associated with HF incidence, such as coronary heart disease (HR: 5.75; 95% CI: 5.17-6.40), history of acute myocardial infarct (HR: 7.40; 95% CI: 6.58-8.34), cardiomyopathy (HR: 13.5; 95% CI: 10.6-17.1), and diabetes (HR: 3.34; 95% CI: 2.95-3.79).

Supplemental Table 3 shows the output of the multivariable competing risk regression model based on the 15 most important variables in HF prediction from the random forest model within the training data set (Supplemental Figure 2). Some of the key characteristics such as BMI (HR: 1.06; 95% CI: 1.05-1.07), age (HR: 1.07; 95% CI: 1.06-1.08), heart rate (HR: 1.02; 95% CI: 1.02-1.03), and systolic blood pressure (HR: 1.01; 95% CI: 1.01-1.01) were shown to be significantly associated with HF incidence. Malignant neoplasms of lymphoid, hematopoietic, and related tissue were also shown to be significantly associated with HF incidence (HR: 3.15; 95% CI: 2.69-3.68). A simplified regression model was then constructed after checking for collinearity and replacement of some key characteristics. Cholesterol level (total cholesterol, high-density lipoprotein, and low-density lipoprotein cholesterol), blood pressure (both systolic and diastolic), and medication were removed from the model, to improve simplicity for EMR and primary care use. Instead, diabetes (HR: 1.31; 95% CI: 1.00-1.70), hypertension, cancer types, and duration of cancer diagnoses were introduced into the simplified model (Supplemental Table 3). The calibration slope was 0.715 (95% CI: 0.621-0.809) in predicting 10-year HF incidence in the testing data set.

### Internal validation of the CHERISH risk score

**Figure 1:**
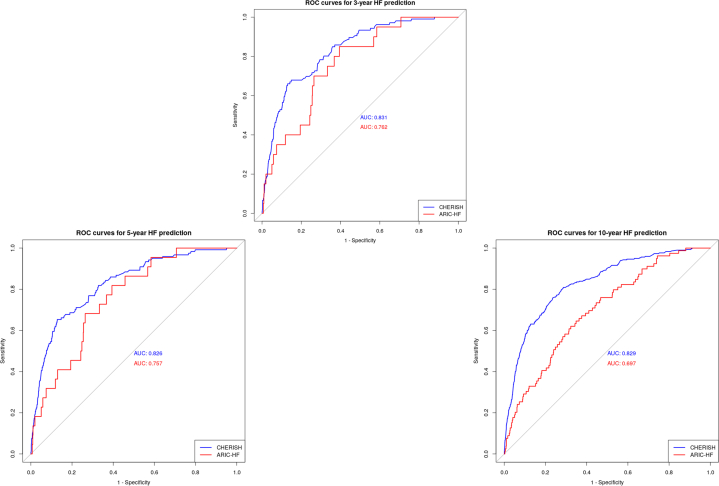
ROC Curves for HF Prediction in the Testing Data Set This figure compares the ROC curves of the CHERISH and ARIC-HF models in predicting 3-, 5-, and 10-year HF incidence among cancer survivors in the UK Biobank validation cohort. CHERISH consistently demonstrated superior discriminative performance across all time points, with a 10-year AUC of 0.83 (95% CI: 0.81-0.85) vs 0.70 (95% CI: 0.64-0.76; *P* < 0.001) for ARIC-HF. These findings support CHERISH as a robust tool for long-term incident HF risk stratification in survivorship care. ARIC = Atherosclerotic Risk In Community studies; AUC = area under the curve; CHERISH = cancer-specific HF prediction from electronic medical records in survivor health care; HF = heart failure; ROC = receiver operating characteristic.

**Figure 2:**
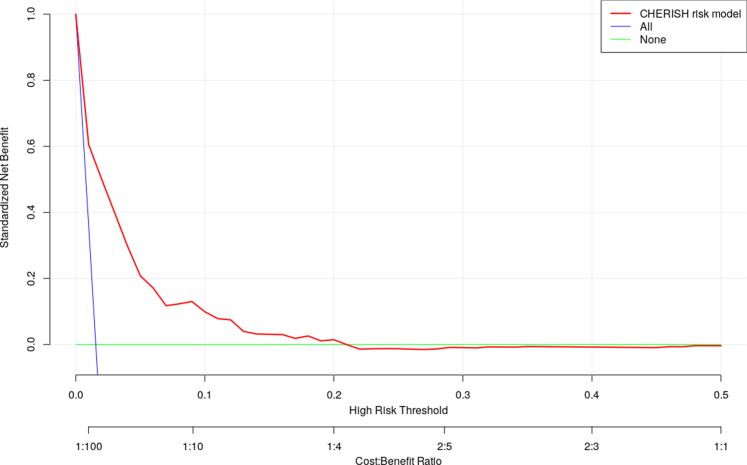
Comparison of Net Benefit Between CHERISH and Default Strategies CHERISH risk score was above both “treat all” and “treat none” across a meaningful range of threshold probabilities (roughly between 0.02 and 0.2), indicating improvement in clinical net benefit. Abbreviations as in Figure 1.

The newly developed CHERISH risk score was then calculated in the UK Biobank testing data set based on the formula in Supplemental Table 4 and the predictive performance is shown in Table 3. The CHERISH 3-year model yielded an overall AUC of 0.83 (95% CI: 0.79-0.87) in predicting 3-year HF incidence, with a sensitivity of 68% and specificity of 85% at its most ideal cutoff value (CHERISH = 3%). A default sensitivity of 80% for the model resulted in a specificity of 69%, whereas 90% sensitivity provided 53% specificity. Figure 1 demonstrates the ROC curves of the CHERISH and ARIC-HF in predicting 3-, 5-, and 10-year HF incidence. Specifically, the CHERISH (AUC: 0.83; 95% CI 0.81-0.85) performed significantly better than ARIC-HF (AUC: 0.70; 95% CI 0.64-0.76; *P* < 0.001) in predicting 10-year HF incidence. The Brier scores were also lower in the CHERISH risk score than the ARIC-HF in all 3 time points. Figure 2 compares the net benefit between the CHERISH risk score and default strategies (treat all or treat none). The CHERISH risk score was shown to provide clinical utility for predicting HF incidence in cancer survivors, especially when the threshold probability for action is between 2% and 15%.

**Table 3:** Prediction of HF Incidence in the UK Biobank Testing Data Set

	3-year HF			5-year HF			10-year HF		
	CHERISH	ARIC-HF	*P* Value	CHERISH	ARIC-HF	*P* Value	CHERISH	ARIC-HF	*P* Value
AUC (95% CI)	0.83 (0.79-0.87)	0.76 (0.67-0.85)	**0.023**	0.83 (0.79-0.86)	0.76 (0.67-0.84)	0.069	0.83 (0.81-0.85)	0.70 (0.64-0.76)	**<0.001**
Brier score	0.014	0.026		0.016	0.029		0.036	0.081	
Sensitivity (ideal cutoff value)	67.92%	60.58%		65.29%	60.61%		75.84%	62.03%	
Specificity (ideal cutoff value)	85.08%	85.00%		87.17%	81.82%		76.46%	67.72%	
Specificity at 80% sensitivity	68.56%	63.23%		67.59%	60.61%		71.89%	44.91%	
Specificity at 90% sensitivity	53.35%	43.12%		47.03%	43.24%		50.22%	30.70%	

Ideal cutoff (CHERISH = 3%) defining survivors with high-risk of HF was determined using Youden index. **Bold** values indicate statistical significance (*P* < 0.05).

AUC = area under the curve; CHERISH = cancer-specific heart failure prediction from electronic medical record in survivor health care; ARIC-HF = heart failure risk score from the Atherosclerotic Risk in Communities study; other abbreviations as in Table 1.

### External validation of the CHERISH risk score

External validation was then performed by applying the CHERISH risk score on cancer survivors in Ontario, Canada (n = 446,096) (Supplemental Figure 1B). Residents' baseline characteristics, stratified via cancer types, are reported in Table 2 and Supplemental Table 5. Overall, the cancer survivors from this cohort were older (mean age 67 years) and the prevalences of comorbidities were higher (eg, diabetes: 23%; hypertension: 59%) than the UK Biobank cohort. The mean 10-year CHERISH risk score in this population was reported to be 10.4 ± 13.8%.

**Figure 3:**
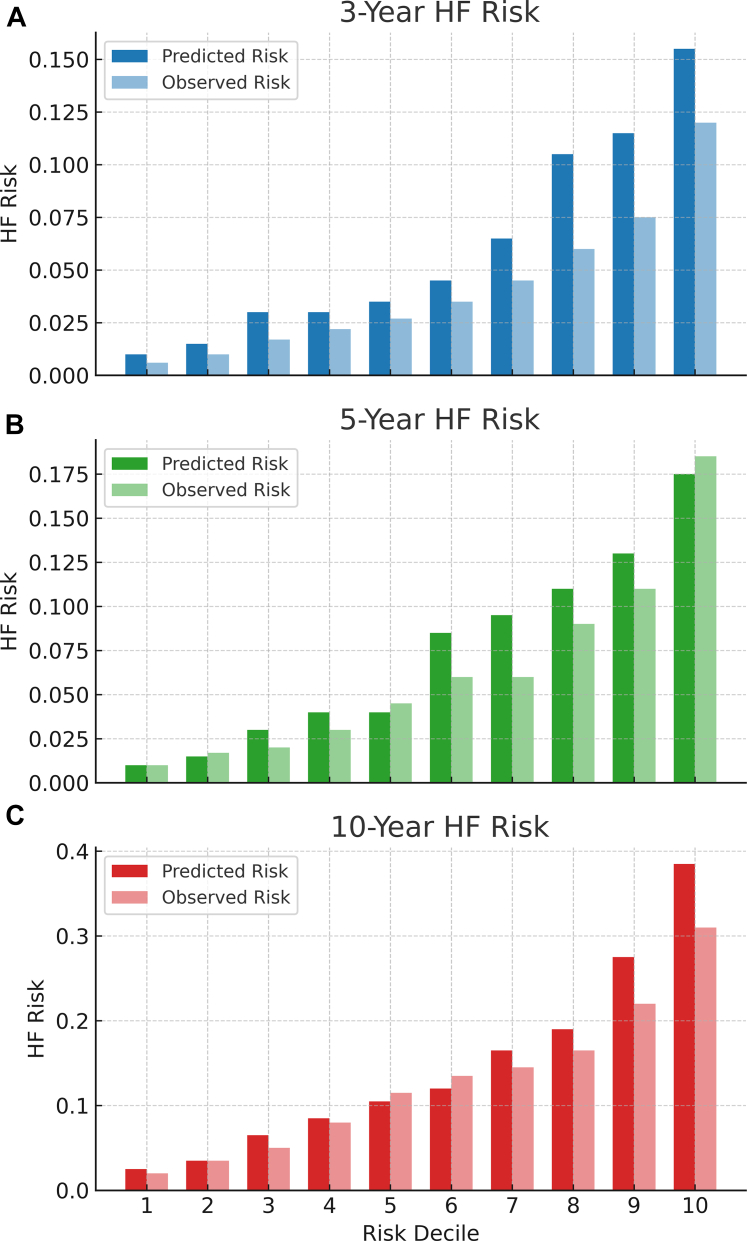
Observed and Predicted HF Incidence by CHERISH in Deciles This figure illustrates the observed vs predicted 10-year incident HF risk across deciles of the CHERISH risk score among cancer survivors in the Ontario administrative database. Calibration was assessed by comparing model-predicted cumulative incidence against observed HF events in each decile. The CHERISH model showed good agreement across the risk spectrum, with better calibration in the mid-to-high deciles. Despite a modest reduction in AUC (0.707), the score maintained robust performance in a real-world, high-risk population, highlighting its potential for scalable implementation in large health systems. Abbreviations as in Figure 1.

Table 4 shows the accuracy of the CHERISH risk score in predicting 3-, 5-, and 10-year HF incidence within the Ontario residents with history of cancer diagnoses. Overall, the CHERISH risk score showed optimal performance in predicting 3-year HF incidence, with an AUC of 0.72, with a sensitivity of 70% and specificity of 63% at its ideal cutoff. In comparison, within the *subpopulation* of 7,921 survivors with complete primary care data, the ARIC-HF showed better performance, with an AUC of 0.75 (95% CI: 0.74-0.77; *P* < 0.001), than the CHERISH risk score (AUC: 0.72; 95% CI: 0.70-0.74). Figure 3 visualizes the calibration of the CHERISH risk score in the total Ontario population, stratifying based on the deciles. Overall, the model showed better calibration at predicting 10-year incident HF risk.

**Table 4:** Predictive Accuracy of CHERISH Risk Score in HF Incidence Among the Ontario Administrative Database

	3-year HF Incidence	5-year HF Incidence	10-year HF Incidence
AUC	0.719	0.717	0.707
Sensitivity (at ideal cutoff)	0.70	0.76	0.77
Specificity (at ideal cutoff)	0.63	0.57	0.59
Specificity (at 80% sensitivity)	0.52	0.53	0.56
Specificity (at 90% sensitivity)	0.37	0.39	0.41

Ideal cutoff (CHERISH = 3%) defining survivors with high-risk of HF was determined using Youden index.

Abbreviations as in Tables 1 and 3.

## Discussion

In this large-scale study, we developed and externally validated the CHERISH risk score for HF in cancer survivors in a manner that allows automated use in EMRs and large health systems. The model showed good discrimination, and superior predictive performance compared to the existing ARIC-HF tool for assessing incident HF risk in cancer survivors. Our findings not only confirmed the elevated risk for HF among cancer survivors but also highlighted particularly high-risk groups, specifically those with hematological malignancies, respiratory cancers, and urinary tract cancers. The CHERISH risk score, incorporating 11 readily available clinical variables, achieved robust predictive accuracy with an AUC of 0.83 in internal validation and 0.71 in external validation. Notably, the model maintained strong predictive performance over a lengthy 10-year period, suggesting its utility as a practical tool for long-term cardiovascular risk stratification in cancer survivors (Central Illustration).

**Central Illustration:**
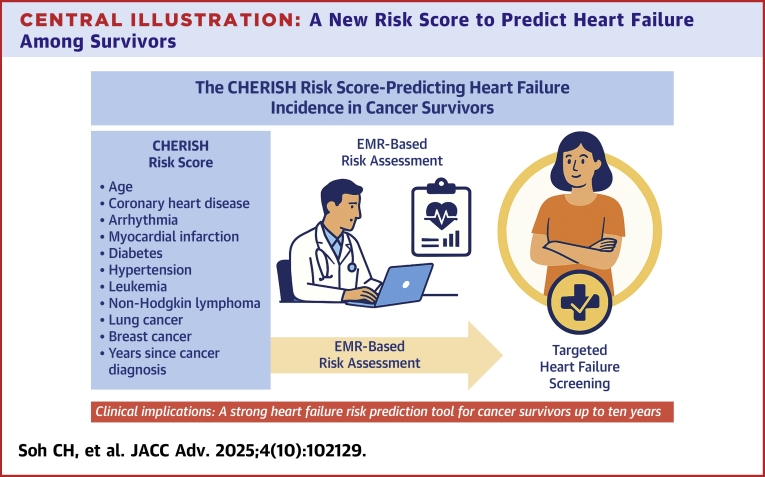
A New Risk Score to Predict Heart Failure Among Survivors This illustration summarizes the CHERISH risk score, a novel tool designed to predict heart failure in cancer survivors. The score incorporates 11 readily available clinical variables, which enables EMR-based incident HF risk assessment at the point of primary care without requiring additional testing or treatment history. The illustration highlights the pathway from cancer survivorship to targeted HF screening, supporting earlier identification of high-risk individuals and proactive cardiovascular care in routine clinical practice. CHERISH = cancer-specific HF prediction from electronic medical records in survivor health care; EMR = electronic medical record.

### Cancer treatments and the subsequent HF occurrence

Among the cancer types analyzed in our study, hematological malignancies, particularly lymphoid and hematopoietic neoplasms, demonstrated the strongest association with HF incidence. This finding corroborates previous population-based cohort studies that reported elevated risks of HF and cardiomyopathy among survivors of non-Hodgkin lymphoma, leukemia, and multiple myeloma during long-term follow-up (>5 years).^16^ The heightened cardiovascular risk in these populations may in part stem from chemotherapy protocols that typically involve anthracycline-based chemotherapy with or without radiotherapy, particularly in non-Hodgkin lymphoma and acute myeloid leukemia, 2 of the most prevalent hematological malignancies.^17^ These findings persist despite substantial efforts over recent decades to mitigate anthracycline-based risk, either through dose reduction or by developing alternative therapeutic agents that maintain treatment efficacy while reducing cardiovascular complications.^18^ Our competing risk regression analyses using the UK Biobank training data set further reinforced these findings, particularly highlighting the significantly elevated incident HF risk among non-Hodgkin's lymphoma survivors. These results suggest ongoing importance of heightened awareness of the incident HF risk in this especially vulnerable patient population.

Breast and lung cancers represent another major category of malignancies associated with the elevated cardiovascular risk. These cancers are less likely to be treated with anthracyclines, and although other agents (eg, trastuzumab) have been associated with cardiotoxicity,^19^ this is generally mild and reversible.^20^^,^^21^ Notably, our analysis revealed no association between breast cancer and HF incidence. This counterintuitive finding might be attributed to several factors: first, the robust cardiac monitoring protocols routinely implemented in breast cancer care, allowing early detection and management of cardiac dysfunction^22^; second, the increasing use of cardioprotective strategies in breast cancer treatment regimens; and third, a potential "healthy survivor effect" in the UK Biobank cohort—potentially a selected population with better overall health status and access to care.^23^ The risk of a “healthy survivor effect” is inherent in a study design that does not follow all cancer survivors at baseline, but instead assesses them at intervals during follow-up (and thereby replicates decision-making when a cancer survivor is reviewed in primary care).

### Cardio-oncology risk assessment

The primary mission of cardio-oncology practice was to minimize cancer therapy–related cardiac dysfunction risk while ensuring optimal cancer treatment.^24^ The growing number of cancer survivors has required an extension of the scope of cardio-oncology to the population of long-term survivors. The HFA-ICOS tool was specifically designed to streamline the process of cardioprotection at the time of chemotherapy, and has demonstrated effectiveness in predicting cardiovascular diseases across multiple real-world validation studies.^25^^,^^26^ However, the cardiovascular risks associated with cancer therapy often extend well beyond the acute treatment phase. This creates a significant challenge in the long-term risk assessment, as post-treatment cardiovascular care typically transitions to primary care physicians.^27^ The HFA-ICOS tool has not been validated in this setting, and is compromised by the limited availability of cancer treatment data and the need for specialized cardiac assessments, including biomarker measurements and echocardiographic data.

Using a random forest algorithm, our study identified several key predictors of HF incidence among cancer survivors, including traditional cardiovascular risk factors such as BMI, age, cholesterol levels, and various cardiovascular comorbidities. These findings align with the predictive factors incorporated in the multiple HF risk assessment tools.^28^ However, our initial machine learning-based regression model faced similar implementation challenges as the HFA-ICOS tool, particularly the requirement for laboratory testing to obtain cholesterol measurements. To address this limitation, we developed the simplified CHERISH model, which relies solely on clinical variables that can be easily obtained in a primary care consultation or by automated extraction from EMRs or administrative data sets. This simplified version maintains robust predictive performance while eliminating the need for additional laboratory testing, making it particularly suitable for routine use in primary care settings where long-term cardiovascular risk management typically occurs. Clinical risk scores may show superior accuracy in predicting incident HF, but their reliance on detailed clinical variables, many of which are not readily available in the EMR, limits their feasibility for large-scale implementation. This limitation was evident in this study, where the sample size eligible for ARIC-HF calculation dropped from 446,096 to just 7,921 due to missing data. The CHERISH risk score thus bridges an important gap in current practice by providing an accessible, scalable tool for ongoing cardiovascular risk assessment in cancer survivors using widely available clinical data. However, its omission of specific cancer treatment variables limits its utility for personalized risk prediction, particularly given the well-established links between these therapies and HF. As such, CHERISH should be viewed not as a comprehensive individualized risk tool, but rather as an EMR–embedded screening mechanism or the first point-of-contact in primary care to flag higher-risk individuals who may benefit from further cardiovascular evaluation. In this context, prioritizing sensitivity over specificity may be appropriate. More detailed clinical risk scores, incorporating treatment exposures and imaging biomarkers, could then be applied to patients identified through CHERISH as a prelude to targeted interventions or diagnostic testing (eg, echocardiography).

The substantial reduction in the CHERISH risk score's discriminative performance in the external validation cohort (AUC: 0.707) compared to internal validation (AUC: 0.829) is likely attributable to notable differences in baseline characteristics between the UK Biobank and Ontario populations. Ontario residents were, on average, older and exhibited substantially higher prevalence of cardiovascular comorbidities, including coronary heart disease (32.7% vs 5.2%) and arrhythmia (23.8% vs 2.2%), indicating a more vulnerable population with a higher baseline risk of HF. Given that CHERISH was developed in a healthier cohort with a lower underlying cardiovascular risk, the model may have systematically underestimated the absolute risk in the external population. This was particularly evident when applying CHERISH to predict 3- and 5-year incident HF risk in the Ontario cohort, where risk overestimation was observed. These findings highlight the importance of recalibrating the model when used in populations with differing baseline hazard rates. Nevertheless, the CHERISH score maintained good calibration across risk deciles, supporting its robustness and potential utility in diverse clinical settings. Future applications should incorporate population-specific recalibration, especially when implementing the model in broader or more clinically representative populations with a higher prevalence of cardiovascular comorbidities, to ensure accurate risk estimation.

### Study limitations

Although CHERISH represents the first risk assessment tool in long-term cancer survivors, suitable for application at the first point-of-contact without additional testing, there are several important limitations. First, the lack of treatment data has resulted in our inability to calculate the HFA-ICOS as a comparator tool.^29^ Second, our reliance on ICD-10 codes in the UK Biobank database for disease classification presents potential limitations. This coding system primarily captures hospital-based diagnoses, potentially missing conditions diagnosed and managed exclusively in primary care settings, leading to possible underestimation of disease prevalence. This limitation is particularly relevant for conditions commonly managed inprimary care, such as early-stage HF or mild cardiovascular complications, which may have led to underperformance in the external validation.^30^ Third, although the simplified nature of CHERISH risk score enhances its practical utility, the limited number of predictive features included may impact its performance in certain patient subgroups. In addition, the UK Biobank cohort is known for “healthy volunteer” selection bias, with participants typically being healthier, better educated, and having fewer self-reported health conditions compared to the general population.^23^ This likely results in a lower baseline risk and fewer cardiovascular events, potentially leading to overly optimistic model performance. Lastly, although we acknowledge the potential bias introduced during a complete case analysis, it was selected to prioritize model simplicity, robustness, and transparency. These are critical considerations when developing and externally validating new clinical prediction models. Although multiple imputation is a common strategy for handling missing data, it may introduce complexity and require assumptions about the missingness mechanism, which, if incorrect, can inadvertently introduce bias and misinformation, potentially affecting the generalizability and predictive accuracy of the final risk model.^31^ Given our goal of external validation and clinical utility, the choice of complete case analysis ensures clarity, reduces the risk of misspecification bias, and supports the clinical interpretability of the model. Future research should evaluate the tool's impact on clinical decision-making and its ability to improve personalized care outcomes, including its cost-effectiveness and potential to reduce cardiovascular complications through early intervention.

## Conclusions

We have developed and validated the CHERISH risk score, a novel incident HF risk assessment tool specifically designed for cancer survivors, demonstrating robust predictive performance in both internal and external validation cohorts. The tool shows particularly strong accuracy in predicting HF incidence within a 3-year timeframe, while maintaining good predictive performance up to 10 years. Unlike existing risk assessment tools, the CHERISH risk score enables rapid risk stratification at the first point-of-contact without requiring additional pathology testing or detailed cancer treatment history, addressing a critical gap in current clinical practice. This accessibility makes CHERISH potentially valuable in primary care settings, where most long-term cancer survivor care occurs. The tool's ability to identify high-risk individuals early in their survivorship journey could facilitate timely initiation of cardioprotective strategies and more targeted monitoring protocols. Future studies should focus on examining the role of CHERISH in guiding treatment to reduce cardiovascular morbidity among cancer survivors.

Perspectives **COMPETENCY IN MEDICAL KNOWLEDGE OR COMPETENCY IN PATIENT CARE:** The CHERISH risk score enables primary care physicians to stratify long-term cancer survivors by incident HF risk at the first clinical contact, without the need for laboratory testing or detailed cancer treatment, supporting proactive longitudinal cardiovascular care in cancer survivors.

**TRANSLATIONAL OUTLOOK:** Prospective studies should assess CHERISH's impact on clinical decision-making, cost-effectiveness, and long-term cardiovascular morbidity reduction among diverse survivor populations.

## Funding support and author disclosures

Dr Marwick is supported by an Investigator grant from the 10.13039/501100000925National Health and Medical Research Council, Canberra, Australia (2008129). All other authors have reported that they have no relationships relevant to the contents of this paper to disclose.
