# Supplementary material for: Development of an Electronic Medical Record–Based Score for Heart Failure Prediction in Cancer Survivors
Source: JACC Adv. 2025 Sep 11;4(10):102129. doi: 10.1016/j.jacadv.2025.102129 (PMC12791888; doi:10.1016/j.jacadv.2025.102129)
Supplement: Supplementary data [file mmc1.docx]

Supplemental Material: TRIPOD checklist - Prediction Model Development.

| **Section/Topic** | **Item** | **Checklist Item** | **Page** |
| --- | --- | --- | --- |
| **Title and abstract** | | | |
| Title | 1 | Identify the study as developing and/or validating a multivariable prediction model, the target population, and the outcome to be predicted. | 1 |
| Abstract | 2 | Provide a summary of objectives, study design, setting, participants, sample size, predictors, outcome, statistical analysis, results, and conclusions. | 2 |
| **Introduction** | | | |
| Background and objectives | 3a | Explain the medical context (including whether diagnostic or prognostic) and rationale for developing or validating the multivariable prediction model, including references to existing models. | 5 |
|  | 3b | Specify the objectives, including whether the study describes the development or validation of the model or both. | 5 |
| **Methods** | | | |
| Source of data | 4a | Describe the study design or source of data (e.g., randomized trial, cohort, or registry data), separately for the development and validation data sets, if applicable. | 5-6 |
|  | 4b | Specify the key study dates, including start of accrual; end of accrual; and, if applicable, end of follow-up. | 5-6 |
| Participants | 5a | Specify key elements of the study setting (e.g., primary care, secondary care, general population) including number and location of centres. | 5-6 |
|  | 5b | Describe eligibility criteria for participants. | 6 |
|  | 5c | Give details of treatments received, if relevant. | N/A |
| Outcome | 6a | Clearly define the outcome that is predicted by the prediction model, including how and when assessed. | 7 |
|  | 6b | Report any actions to blind assessment of the outcome to be predicted. | N/A |
| Predictors | 7a | Clearly define all predictors used in developing or validating the multivariable prediction model, including how and when they were measured. | 6-9 |
|  | 7b | Report any actions to blind assessment of predictors for the outcome and other predictors. | N/A |
| Sample size | 8 | Explain how the study size was arrived at. | 5-6 |
| Missing data | 9 | Describe how missing data were handled (e.g., complete-case analysis, single imputation, multiple imputation) with details of any imputation method. | 9 |
| Statistical analysis methods | 10a | Describe how predictors were handled in the analyses. | 8-9 |
|  | 10b | Specify type of model, all model-building procedures (including any predictor selection), and method for internal validation. | 8-9 |
|  | 10d | Specify all measures used to assess model performance and, if relevant, to compare multiple models. | 8-9 |
| Risk groups | 11 | Provide details on how risk groups were created, if done. | N/A |
| **Results** | | | |
| Participants | 13a | Describe the flow of participants through the study, including the number of participants with and without the outcome and, if applicable, a summary of the follow-up time. A diagram may be helpful. | 10; Supp. Fig 1 |
|  | 13b | Describe the characteristics of the participants (basic demographics, clinical features, available predictors), including the number of participants with missing data for predictors and outcome. | 10 |
| Model development | 14a | Specify the number of participants and outcome events in each analysis. | 10 |
|  | 14b | If done, report the unadjusted association between each candidate predictor and outcome. | 10; Supp Table 2 |
| Model specification | 15a | Present the full prediction model to allow predictions for individuals (i.e., all regression coefficients, and model intercept or baseline survival at a given time point). | 10; Supp Table 3 |
|  | 15b | Explain how to the use the prediction model. | 9; Supp Table 3 |
| Model performance | 16 | Report performance measures (with CIs) for the prediction model. | 11; Table 3 |
| **Discussion** | | | |
| Limitations | 18 | Discuss any limitations of the study (such as nonrepresentative sample, few events per predictor, missing data). | 16 |
| Interpretation | 19b | Give an overall interpretation of the results, considering objectives, limitations, and results from similar studies, and other relevant evidence. | 12-16 |
| Implications | 20 | Discuss the potential clinical use of the model and implications for future research. | 16; 18 |
| **Other information** | | | |
| Supplemental information | 21 | Provide information about the availability of Supplemental resources, such as study protocol, Web calculator, and data sets. | 9 |
| Funding | 22 | Give the source of funding and the role of the funders for the present study. | 1 |

Supplemental Table 1: Comparison of Model Fit Using Linear vs Spline Terms for Continuous Predictors.

|  | AIC | | |
| --- | --- | --- | --- |
|  | Linear |  | Spline |
| Age | 4619.3 |  | **4618.5** |
| Years since cancer diagnosis | 4624 |  | **4620.4** |
| Body mass index | **4608.8** |  | 4611.7 |
| Systolic blood pressure | **4320.7** |  | 4325.7 |
| Heart rate | **4317.4** |  | 4320.3 |
| Total cholesterol | **4221.8** |  | 4227.2 |

AIC: Akaike Information Criterion.

Supplemental Table 2: Variables included in the machine learning model and their corresponding importance in HF prediction and hazard ratio in univariable competing risk regression.

| Variables | Mean decrease in Gini coefficient | Hazard Ratio  (95% CI) |
| --- | --- | --- |
| Body mass index | 63.569 | **1.08 (1.07-1.09)** |
| Age | 50.975 | **1.10 (1.09-1.11)** |
| Low-density lipoprotein cholesterol | 47.838 | **0.68 (0.64-0.72)** |
| Heart rate | 47.547 | **1.01 (1.01-1.02)** |
| Total cholesterol | 46.862 | **0.72 (0.69-0.75)** |
| High-density lipoprotein cholesterol | 46.798 | **0.40 (0.35-0.46)** |
| Systolic blood pressure | 45.307 | **1.01 (1.01-1.02)** |
| Diastolic blood pressure | 42.476 | 1.00 (1.00-1.01) |
| Coronary heart disease | 22.910 | **5.75 (5.17-6.40)** |
| Blood pressure medication | 16.438 | **3.05 (2.80-3.34)** |
| Arrhythmia | 15.769 | **6.63 (5.83-7.54)** |
| History of acute myocardial infarct | 15.084 | **7.40 (6.58-8.34)** |
| Malignant neoplasms of lymphoid, haematopoietic and related tissue | 11.611 | **3.08 (2.67-3.55)** |
| Smoking status – Current smoker | 11.390 | **2.04 (1.77-2.34)** |
| Cholesterol medication | 10.067 | **3.02 (2.76-3.30)** |
| Cardiomyopathy | 7.674 | **13.5 (10.6-17.1)** |
| Malignant neoplasms of breast | 7.459 | **0.82 (0.72-0.93)** |
| Alcohol consumption | 7.328 | **1.35 (1.04-1.75)** |
| Valvular heart disease | 7.203 | **8.90 (7.54-10.5)** |
| Sex - Male | 7.145 | **2.09 (1.92-2.29)** |
| Malignant neoplasms of ill-defined and unspecified sites | 6.693 | **2.68 (2.27-3.15)** |
| Angina | 5.967 | **4.71 (4.11-5.41)** |
| Malignant neoplasms of digestive organs | 5.484 | **1.75 (1.51-2.01)** |
| Malignant neoplasms of male genital organs | 5.434 | **1.56 (1.35-1.81)** |
| Malignant neoplasms of urinary tract | 5.237 | **2.18 (1.85-2.57)** |
| Diabetes | 4.303 | **3.34 (2.95-3.79)** |
| Malignant neoplasms of female genital organs | 3.404 | 0.90 (0.70-1.16) |
| Ethnicity - White | 3.276 | 0.62 (0.32-1.18) |
| Malignant neoplasms of respiratory and intrathoracic organs | 3.239 | **3.08 (2.48-3.83)** |
| Malignant neoplasms of lip, oral cavity and pharynx | 2.438 | 1.36 (0.94-1.98) |
| Chronic kidney disease | 2.335 | **8.82 (6.90-11.3)** |
| Malignant neoplasms of multiple sites | 1.761 | 1.09 (0.72-1.66) |
| Insulin injection | 1.370 | **4.84 (3.88-6.03)** |
| Malignant neoplasms of eye, brain and other parts of central nervous system | 1.161 | 1.41 (0.78-2.55) |
| Malignant neoplasms of thyroid and other endocrine glands | 1.040 | 1.32 (0.81-2.16) |
| Malignant neoplasms of bone and articular cartilage | 0.480 | 1.34 (0.50-3.57) |

Supplemental Table 3: Multivariable competing risk regression on the association between HF risk factors and HF incidence in the training dataset.

|  | Initial model | |  | Simplified (CHERISH) model | |
| --- | --- | --- | --- | --- | --- |
|  | HR (95% CI) | p-value |  | HR (95% CI) | p-value |
| Body mass index | 1.055 (1.045-1.066) | <0.001 |  | - | - |
| Age | 1.073 (1.062-1.084) | <0.001 |  | 1.043 (1.018-1.069) | 0.001 |
| Low-density lipoprotein cholesterol | 1.152 (0.843-1.574) | 0.3751 |  | - | - |
| Heart rate | 1.021 (1.017-1.025) | <0.001 |  | - | - |
| Total cholesterol | 0.777 (0.606-0.996) | 0.0461 |  | - | - |
| High-density lipoprotein cholesterol | 1.090 (0.863-1.377) | 0.4685 |  | - | - |
| Systolic blood pressure | 1.009 (1.006-1.013) | <0.001 |  | - | - |
| Diastolic blood pressure | 0.981 (0.974-0.987) | <0.001 |  | - | - |
| Coronary heart disease | 2.227 (1.942-2.554) | <0.001 |  | 7.009 (5.186-9.504) | <0.001 |
| Blood pressure medication | 1.464 (1.304-1.644) | <0.001 |  | - | - |
| Arrhythmia | 3.909 (3.377-4.524) | <0.001 |  | 3.004 (1.996-4.406) | <0.001 |
| Diabetes | - | - |  | 1.467 (1.092-1.955) | 0.01 |
| Hypertension | - | - |  | 1.500 (1.100-2.065) | 0.012 |
| History of acute myocardial infarct | 3.271 (2.826-3.787) | <0.001 |  | 1.147 (0.802-1.618) | 0.444 |
| Malignant neoplasms of lymphoid, haematopoietic and related tissue | 3.145 (2.691-3.677) | <0.001 |  | - | - |
| Smoking status |  |  |  |  |  |
| Never smoke | Ref | - |  | - | - |
| Previous smoker | 1.269 (1.141-1.413) | <0.001 |  | - | - |
| Current smoker | 2.027 (1.734-2.369) | <0.001 |  | - | - |
| Cholesterol medication | 1.014 (0.887-1.159) | 0.8393 |  | - | - |
| Non-Hodgkin’s lymphoma | - | - |  | 2.299 (1.527-3.366) | <0.001 |
| Leukaemia | - | - |  | 1.135 (0.551-2.084) | 0.707 |
| Lung cancer | - | - |  | 1.074 (0.596-1.798) | 0.798 |
| Breast cancer | - | - |  | 0.673 (0.478-0.931) | 0.020 |
| Years since cancer diagnosis | - | - |  | 0.999 (0.981-1.019) | 0.932 |

HF: Heart failure; OR: Odds ratio; CI: Confidence interval; HDL: High-density lipoprotein; LDL: Low-density lipoprotein.

Supplemental Table 4: Regression coefficients of the CHERISH model.

|  | 3-year model |  | 5-year model |  | 10-year model |
| --- | --- | --- | --- | --- | --- |
|  | β coefficient |  | β coefficient |  | β coefficient |
| Intercept | -5.48708 |  | -5.60588 |  | -5.08975 |
| Age (46-50y) | -0.92945 |  | -0.64357 |  | -0.28187 |
| Age (51-55y) | -0.10024 |  | 0.11215 |  | 0.01134 |
| Age (56-60y) | 0.27304 |  | 0.40429 |  | 0.37886 |
| Age (61-65y) | 0.10425 |  | 0.28947 |  | 0.16080 |
| Age (66+y) | 0.59877 |  | 0.72749 |  | 0.75402 |
| Coronary heart disease | 1.95583 |  | 2.04530 |  | 1.83850 |
| Hypertension | 0.41765 |  | 0.43525 |  | 0.62276 |
| Arrhythmia | 1.11865 |  | 0.99685 |  | 1.01944 |
| Diabetes | 0.37431 |  | 0.35694 |  | 0.54109 |
| History of acute myocardial infarct | 0.12449 |  | 0.12545 |  | 0.32456 |
| Non-Hodgkin’s lymphoma | 0.83637 |  | 0.74172 |  | 0.86029 |
| Leukaemia | 0.11537 |  | 0.32027 |  | 0.69653 |
| Breast cancer | -0.40601 |  | -0.35667 |  | -0.18977 |
| Lung cancer | 0.05867 |  | 0.31543 |  | 0.37587 |
| Years since cancer dx (2^nd^) | 0.09724 |  | 0.16454 |  | 0.21283 |
| Years since cancer dx (3^rd^) | -0.03647 |  | 0.05746 |  | 0.22134 |
| Years since cancer dx (4^th^) | -0.05758 |  | 0.03352 |  | 0.11086 |
| Years since cancer dx (5^th^) | -0.21041 |  | -0.20658 |  | 0.05191 |
| Years since cancer dx (6-10^th^) | -0.08589 |  | 0.01191 |  | 0.27163 |
| Years since cancer dx (11-15^th^) | 0.25344 |  | 0.34598 |  | 0.51536 |
| Years since cancer dx (16-20^th^) | 0.19688 |  | 0.28112 |  | 0.55433 |
| Years since cancer dx (21^st^ onward) | 0.25978 |  | 0.04682 |  | 0.51308 |

CHERISH: Cancer Heart Evaluation and Risk Inferred from Survivors’ Health record.

*CHERISH model was constructed based on the simplified model.

CHERISH is reported as Y% risk of HF occurrence, where Y = $\frac{XB}{1+XB}*100$

B = β_intercept_ + (β_bmi_ * body mass index) + (β_age_ * age) + (β_LDL cholesterol_ * LDL cholesterol) + (β_heart rate_ * heart rate) + ……

XB = Exponential (B)

Supplemental Table 5a: Baseline characteristics of the Ontario residents included in the external validation.

|  | Total | Lip/Oral/Pharynx | Digestive organs | Respiratory | Bone | Breast | Female genital |
| --- | --- | --- | --- | --- | --- | --- | --- |
|  | N=446,096 | n=11,259 | n=66,674 | n=22,006 | n=609 | n=99,892 | n=39,143 |
| Age at index date, years | 67.87 (12.23) | 65.30 (11.80) | 70.77 (11.95) | 70.01 (10.93) | 60.59 (12.78) | 66.78 (12.13) | 65.89 (12.37) |
| Female, n (%) | 240,667 (53.9%) | 3,835 (34.1%) | 30,831 (46.2%) | 10,802 (49.1%) | *269-273 | 99,345 (99.5%) | *39138-39142 |
| Years since cancer diagnosis, years | 9.06 (8.14) | 9.19 (8.84) | 8.02 (7.50) | 6.44 (7.25) | 14.36 (11.57) | 10.13 (8.35) | 11.38 (9.91) |
| History of malignant neoplasms of respiratory and intrathoracic organs | 23,907 (5.4%) | 121 (1.1%) | 329 (0.5%) | 22,006 (100.0%) | 10 (1.6%) | 225 (0.2%) | 50 (0.1%) |
| History of malignant neoplasms of lymphoid, haematopoietic and related tissue | 39,773 (8.9%) | 116 (1.0%) | 478 (0.7%) | 270 (1.2%) | *1-5 | 439 (0.4%) | 150 (0.4%) |
| History of non-Hodgkin’s lymphoma | 20,526 (4.6%) | 52 (0.5%) | 271 (0.4%) | 165 (0.7%) | *1-5 | 216 (0.2%) | 85 (0.2%) |
| History of leukemia | 11,462 (2.6%) | 29 (0.3%) | 108 (0.2%) | 59 (0.3%) | *1-5 | 89 (0.1%) | 33 (0.1%) |
| History of lung cancer | 19,110 (4.3%) | 36 (0.3%) | 246 (0.4%) | 17,874 (81.2%) | *1-5 | 200 (0.2%) | 42 (0.1%) |
| History of breast cancer | 105,049 (23.5%) | 140 (1.2%) | 1,256 (1.9%) | 797 (3.6%) | 6 (1.0%) | 99,892 (100.0%) | 1,166 (3.0%) |
| Hypertension prior to index | 262,861 (58.9%) | 6,163 (54.7%) | 42,695 (64.0%) | 13,671 (62.1%) | 295 (48.4%) | 53,178 (53.2%) | 22,504 (57.5%) |
| Diabetes prior to index | 100,471 (22.5%) | 2,206 (19.6%) | 18,074 (27.1%) | 4,997 (22.7%) | 129 (21.2%) | 18,023 (18.0%) | 9,053 (23.1%) |
| Acute myocardial infarction prior to index | 32,866 (7.4%) | 855 (7.6%) | 5,840 (8.8%) | 2,303 (10.5%) | 46 (7.6%) | 4,384 (4.4%) | 1,819 (4.6%) |
| Coronary heart disease prior to index | 156,426 (35.1%) | 3,609 (32.1%) | 25,960 (38.9%) | 9,596 (43.6%) | 200 (32.8%) | 27,986 (28.0%) | 10,774 (27.5%) |
| History of arrhythmia prior to index | 104,579 (23.4%) | 2,256 (20.0%) | 17,049 (25.6%) | 5,818 (26.4%) | 107 (17.6%) | 21,494 (21.5%) | 8,027 (20.5%) |
| CHERISH 10-year risk score, % | 10.44 (13.83) | 8.91 (12.72) | 11.13 (13.60) | 14.90 (16.57) | 5.00 (8.74) | 7.12 (10.68) | 8.23 (11.61) |
| ARIC-HF 10-year risk score, % | 7.50 (11.85) | 6.98 (12.89) | 9.55 (13.89) | 9.72 (13.29) | 2.16 (4.97) | 4.39 (8.11) | 4.75 (8.86) |

Index date: Jan 1, 2014. ARIC-HF is calculated in the subpopulation of N=7,921 with complete primary care data.

*Count and proportion are suppressed to minimize risk of patient identification, including back calculation using other reported results.

CHERISH: Cancer HEart RIsk from Survivors’ Health record; ARIC-HF: Heart failure risk score from the Atherosclerotic Risk in Community Study.

Supplemental Table 5b: Baseline characteristics of the Ontario residents included in the external validation (cont.).

|  | Male genital | Urinary | Eye/Brain/CNS | Thyroid/Endocrine | Lymphoid/  Haemotology | Other |
| --- | --- | --- | --- | --- | --- | --- |
|  | n=95,410 | n=28,615 | n=4,761 | n=27,201 | n=40,955 | n=9,571 |
| Age at index date, years | 71.23 (10.26) | 69.96 (11.98) | 60.01 (12.71) | 58.06 (11.35) | 65.82 (12.91) | 66.43 (13.38) |
| Female, n (%) | 12 (0.0%) | 9,246 (32.3%) | 2,271 (47.7%) | 21,525 (79.1%) | 18,899 (46.1%) | 4,490 (46.9%) |
| Years since cancer diagnosis, years | 8.05 (6.22) | 9.15 (8.37) | 12.20 (11.13) | 9.48 (8.31) | 9.15 (8.82) | 7.73 (9.31) |
| History of malignant neoplasms of respiratory and intrathoracic organs | 277 (0.3%) | 162 (0.6%) | 15 (0.3%) | 81 (0.3%) | 566 (1.4%) | 65 (0.7%) |
| History of malignant neoplasms of lymphoid, haematopoietic and related tissue | 576 (0.6%) | 237 (0.8%) | *28-32 | 187 (0.7%) | 40,955 (100.0%) | 160 (1.7%) |
| History of non-Hodgkin’s lymphoma | 334 (0.4%) | 148 (0.5%) | *11-15 | 83 (0.3%) | 19,081 (46.6%) | 75 (0.8%) |
| History of leukemia | 183 (0.2%) | 61 (0.2%) | *3-7 | 25 (0.1%) | 10,805 (26.4%) | 62 (0.6%) |
| History of lung cancer | 152 (0.2%) | 130 (0.5%) | *9-13 | 57 (0.2%) | 314 (0.8%) | 45 (0.5%) |
| History of breast cancer | 37 (0.0%) | 372 (1.3%) | 46 (1.0%) | 415 (1.5%) | 737 (1.8%) | 185 (1.9%) |
| Hypertension prior to index | 62,400 (65.4%) | 20,069 (70.1%) | 2,079 (43.7%) | 12,369 (45.5%) | 21,798 (53.2%) | 5,640 (58.9%) |
| Diabetes prior to index | 23,111 (24.2%) | 8,168 (28.5%) | 778 (16.3%) | 5,108 (18.8%) | 8,781 (21.4%) | 2,043 (21.3%) |
| Acute myocardial infarction prior to index | 9,566 (10.0%) | 3,052 (10.7%) | 226 (4.7%) | 832 (3.1%) | 3,094 (7.6%) | 849 (8.9%) |
| Coronary heart disease prior to index | 40,661 (42.6%) | 12,766 (44.6%) | 1,230 (25.8%) | 6,311 (23.2%) | 13,855 (33.8%) | 3,478 (36.3%) |
| History of arrhythmia prior to index | 23,923 (25.1%) | 7,670 (26.8%) | 883 (18.5%) | 5,549 (20.4%) | 9,415 (23.0%) | 2,388 (25.0%) |
| CHERISH 10-year risk score, % | 12.51 (14.49) | 12.26 (13.73) | 7.69 (12.94) | 7.01 (11.52) | 15.82 (19.09) | 10.53 (12.95) |
| ARIC-HF 10-year risk score, % | 11.15 (13.66) | 10.47 (13.70) | 3.85 (6.82) | 3.16 (7.07) | 7.08 (11.34) | 8.55 (13.71) |

Index date: Jan 1, 2014. ARIC-HF is calculated in the subpopulation of N=7,921 with complete primary care data.

*Count and proportion are suppressed to minimize risk of patient identification, including back calculation using other reported results.

CHERISH: Cancer HEart RIsk from Survivors’ Health record; ARIC-HF: Heart failure risk score from the Atherosclerotic Risk in Community Study.

Supplemental Figure 1: Flowchart of the included participants.


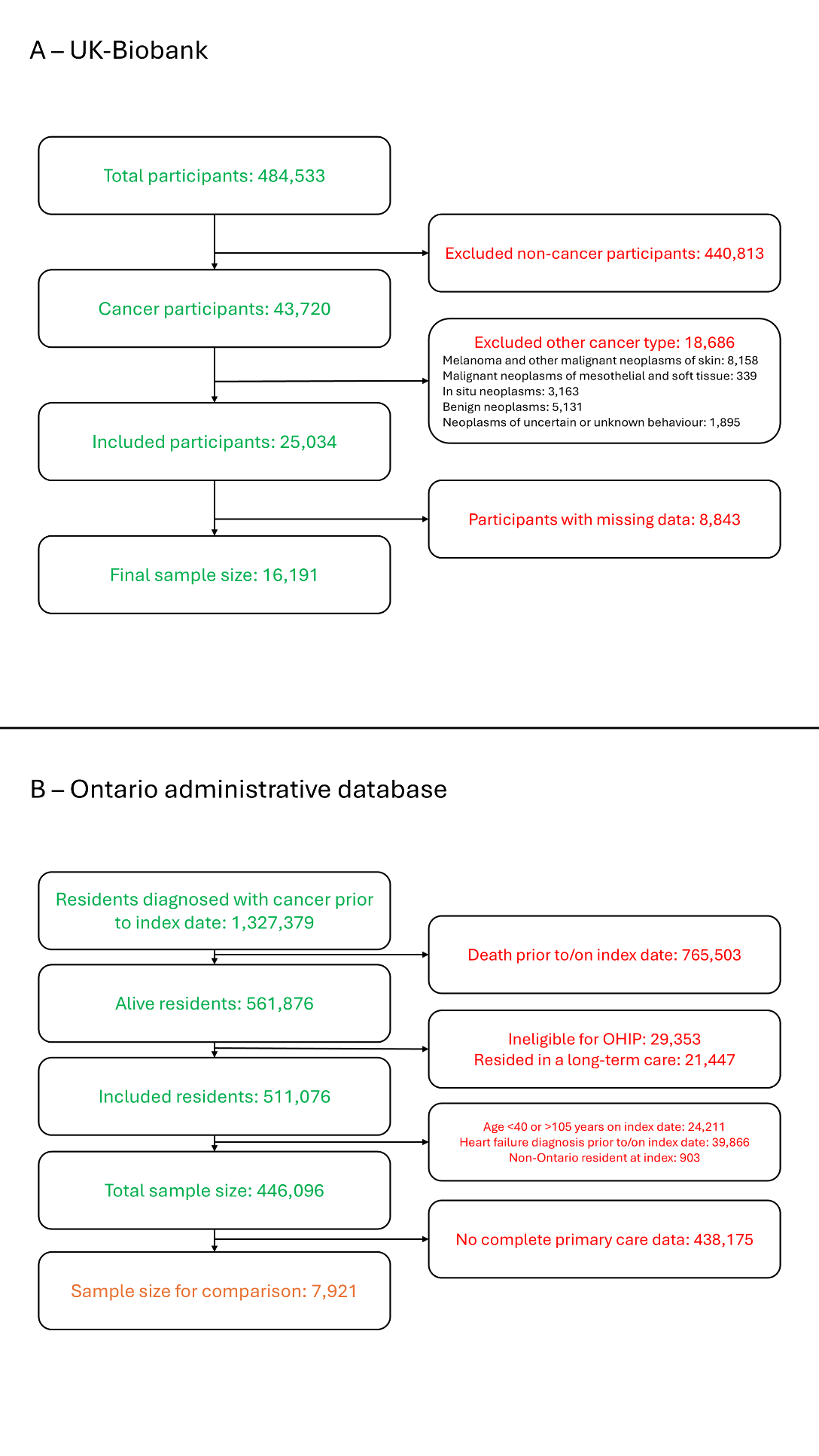


Panel A: Flowchart of participants included from the UK-Biobank database.

Panel B: Flowchart of participants included from the Ontario administrative database. Index date: January 1, 2014.

OHIP: Ontario Health Insurance Plan.

Supplemental Figure 2: Variables that were identified as important in predicting HF incidence using machine learning (mean decrease in Gini coefficient >10).


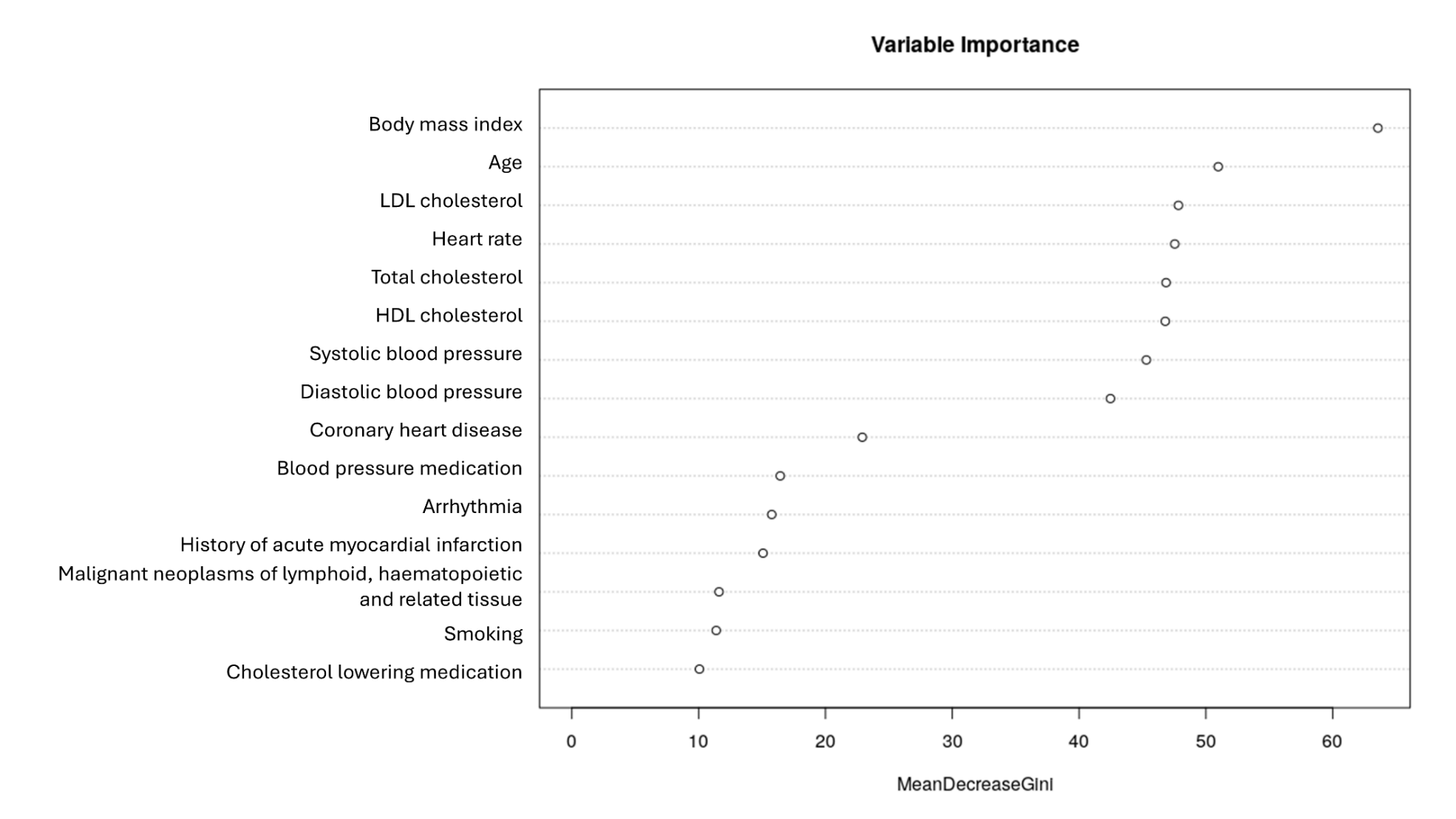


HF: Heart failure; LDL: low-density lipoprotein; HDL: high-density lipoprotein.
